# The Impact of the Vaginal Microbiome and Microbial Metabolites on HIV Transmission at the Female Reproductive Tract Epithelial Barrier

**DOI:** 10.1111/aji.70312

**Published:** 2026-08-18

**Authors:** Brianna Jesaveluk, Paula Ellenberg, Anna C. Hearps, Gilda Tachedjian

**Affiliations:** ^1^ Life Sciences Discipline and Disease Elimination Program Burnet Institute Melbourne Victoria Australia; ^2^ Department of Infectious Diseases Monash University Melbourne Victoria Australia; ^3^ Department of Infectious Diseases University of Melbourne Melbourne Victoria Australia; ^4^ Department of Microbiology and Immunology at the Peter Doherty Institute for Infection and Immunity University of Melbourne Melbourne Victoria Australia; ^5^ Department of Microbiology Monash University Clayton Victoria Australia

**Keywords:** epithelial barrier, female reproductive tract, HIV transmission, lactobacillus, lactic acid, microbiome

## Abstract

Despite major advances in biomedical HIV prevention strategies, almost 50% of the 1.3 million new HIV infections in 2024 occurred in women and girls through heterosexual transmission. Choice in HIV prevention modalities is needed throughout a women's lifespan to increase pre‐exposure prophylaxis uptake; however, there is a paucity of topical, on‐demand options. To establish HIV infection, the virus translocates across the epithelial barrier of the upper and lower female reproductive tract to infect CD4 + HIV target cells in the submucosa. The vaginal microbiome modulates this process. Women colonised with an optimal *Lactobacillus*‐dominated vaginal microbiota are more protected against acquiring HIV compared to women with a non‐optimal vaginal microbiota including bacterial vaginosis (BV), characterised by an overgrowth of diverse anaerobes and a paucity of beneficial lactobacilli. Optimal *Lactobacillus* spp. produce ∼1% lactic acid, which in women with a *Lactobacillus*‐dominant microbiota acidifies the vaginal pH to less than 4.5, inactivates HIV, exerts antimicrobial effects against BV–associated bacteria, directly inhibits inflammatory responses elicited from cervicovaginal epithelial cells and blocks HIV translocation in vitro. Beneficial properties of optimal vaginal lactobacilli and/or lactic acid can potentially be exploited in vaginal delivery strategies to help prevent vaginal dysbiosis and its adverse sexual and reproductive outcomes including HIV.

## 1 HIV Transmission and Prevention in Women

Globally, 45% of all new HIV infections were among women and girls in 2024 [1]. Most HIV infections in women are due to male‐to‐female transmission by penetrative vaginal sex [2]. Providing women with wider choice in pre‐exposure prophylaxis (PrEP) modalities can increase adherence and reduce HIV transmission [3, 4, 5]. Vaginal delivery mechanisms such as gels, intravaginal rings, or films can provide local delivery of drugs such as antiretrovirals for HIV PrEP, but do not provide systemic protection or protect against rectal exposure [6, 7]. There are several advantages to local vaginal delivery that include fewer systemic side effects, discreteness, a large surface area for drug absorption, as well as providing autonomy to self‐administer interventions [6]. On‐demand technologies (e.g., films) are in the development pipeline for sustained vaginal drug delivery that can deliver multiple active agents for use as multipurpose prevention technologies to prevent HIV as well as other sexually transmitted infections (STIs) or to prevent unplanned pregnancy [7, 8, 9, 10]. The composition of the vaginal microbiota is a key modulator of HIV risk [11, 12], with additional consequences for the sexual and reproductive health (SRH) of women [13]. This review aims to explore mechanisms of HIV transmission in the female reproductive tract (FRT) and how novel strategies to optimise the vaginal microbiota and mucosal environment could decrease HIV transmission risk in women.

HIV primarily establishes infection in women through the FRT [14, 15] where the virus penetrates the epithelial barrier to infect immune target cells [16]. The FRT can be broadly divided into the upper and lower FRT where the epithelium is structurally distinct [16]. The upper FRT includes the uterus, ovaries, fallopian tubes and endocervix, which are lined with a single layer of columnar epithelium [14, 16]. The lower FRT includes the ectocervix and vagina, which have multilayered, stratified squamous epithelium [14, 16]. Some upper FRT epithelial tissues such as the fallopian tubes are ciliated, facilitating mucus distribution, which may modulate HIV acquisition risk in the upper FRT [17, 18, 19]. Vaginal mucus serves as a physical barrier against infection, distributing key immune mediators [20] and modulating the growth of vaginal microbiota species [21]. Typically, the FRT epithelium provides a strong physical and immunological barrier against pathogens [14]. Cell‐cell junctions play a key role in the function of the epithelial barrier, with studies in non‐human primates and human vaginal explant tissue showing that HIV particles penetrate the upper layers of an intact epithelial barrier by a paracellular pathway [14, 22, 23]. However, their further migration is blocked by intercellular junctions, which link neighbouring epithelial cells [14, 24].

Heightened FRT epithelial barrier permeability is associated with poorer SRH health outcomes [14, 22, 25, 26, 27, 28]. Disruptions to epithelial barrier and tight junction integrity may be due to physical abrasions and wounds from sexual activity [29, 30]. Increased levels of pro‐inflammatory cytokines and chemokines are frequently associated with FRT barrier disruption [22, 31]. Both commensal and pathogenic microbiota including STI pathogens modulate epithelial barrier integrity, genital ulceration, inflammation, and junctional integrity, which further impacts the transmission risk of pathogens such as HIV [32, 33, 34, 35, 36].

## 2 Mechanisms of HIV Transmission at the FRT Epithelial Barrier

**FIGURE 1:**
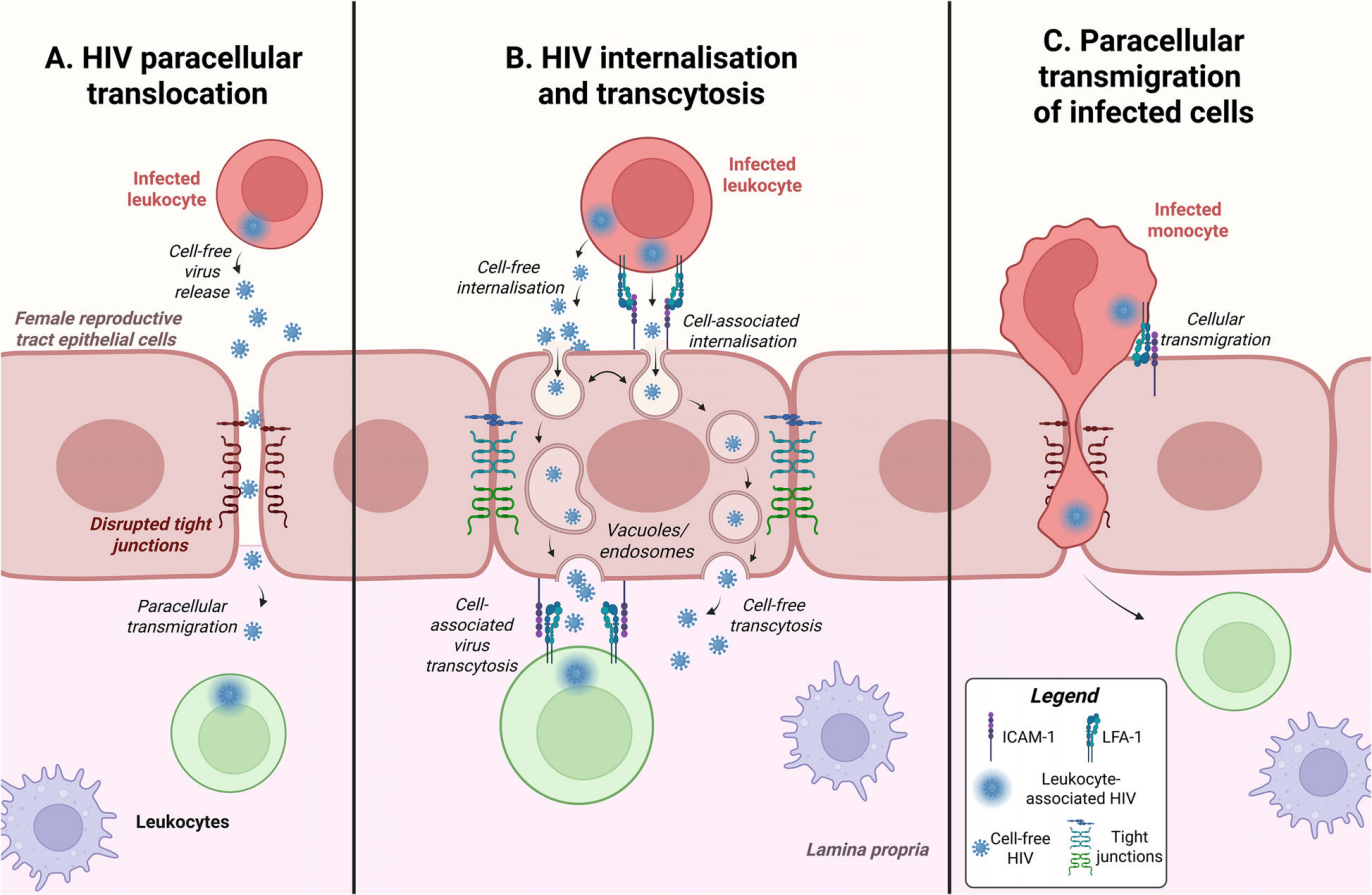
Mechanisms of HIV translocation through cervicovaginal epithelial cells. (A) Cell‐free HIV paracellular translocation, (B) Cell‐free and cell‐associated HIV internalisation and transcytosis, (C) Paracellular transmigration of infected monocytes and cell‐free HIV release or trans‐infection of target cells. Although represented above as a monolayer for simplicity, vaginal and ectocervical epithelia exist as stratified squamous epithelia. HIV virions express cell surface markers including leukocyte function‐associated antigen‐1 (LFA‐1) and intercellular adhesion molecule‐1 (ICAM‐1) derived from host cell membranes. Created with BioRender.com.

HIV must overcome numerous biological barriers in the FRT to establish infection in target cells, including the epithelial tissue, immune mediators, mucus, and other components of cervicovaginal fluid (CVF) including antimicrobial proteins and metabolites [37, 38, 39, 40]. HIV infection can occur throughout the upper and lower FRT [15] although the surface area of the lower FRT is significantly larger [41], increasing potential opportunities for HIV to enter through breaches in the epithelium [41, 42]. HIV present in semen must come into direct contact with HIV target cells typically located in the sub‐epithelium to establish infection [38]. HIV transmission in the FRT is aided by seminal fluid which temporarily neutralises the acidic vaginal environment and enhances viral migration [43, 44]. HIV may migrate between epithelial cells in a process called paracellular translocation (Figure 1A), which requires the virus to overcome tight junctions between adjacent cells [14, 45]. Although cells of the FRT epithelium do not support HIV replication, the virus can be endocytosed from the apical surface, migrate through the cytoplasm, and be released from the basolateral cell membrane, known as transcytosis (Figure 1B) [46, 47]. HIV transcytosis through epithelial tissue has been observed in ex vivo human columnar cervical epithelial explants [48]. Infected cells such as monocytes and macrophages may also translocate through the epithelial barrier and trans‐infect target cells (Figure 1C) [42]. HIV can undergo transcytosis across primary FRT epithelial cells despite an absence of CD4 and coreceptor expression, suggesting other receptors are likely to mediate viral transmission by transcytosis [49].

While cell‐free transcytosis can occur, the process is inefficient, with approximately 0.01%–0.2% of the inoculum undergoing transcytosis in in vitro studies [24, 49, 50]. Seminal fluid contains HIV infected leukocytes including macrophages that could mediate cell‐to‐cell transmission [42, 51, 52], with in vitro evidence suggesting that HIV dissemination may increase by 100‐ to 1000‐fold using cell‐associated mechanisms of transmission [53, 54, 55]. HIV transmission in the FRT is influenced by numerous biological factors, importantly including the composition of the microbiota which colonise the cervicovaginal environment.

## 3 The Microbiome of the Human Body and the FRT

The human body is colonised by commensal bacteria at mucosal and epidermal sites which markedly differ in species composition between sites and between individuals [56]. In the gut, colonisation with diverse commensal bacteria is protective as the microbiota outcompete pathogenic species, produces essential nutrients and is important for homeostatic immune function [57]. In contrast, a vaginal microbiome (VMB) dominated by *Lactobacillus* spp. is considered optimal, whilst a diverse VMB is considered dysbiotic, or non‐optimal. The human VMB is unique in its composition and function and has been identified as playing a key role in SRH [58, 59]. It is distinct from other mammals, including non‐human primates [60], in part due to the deposition of glycogen in the FRT which promotes the growth and metabolism of *Lactobacillus* spp. [60, 61, 62]. *Lactobacilli* are rod‐shaped, Gram positive facultative anaerobic bacteria which acidify the vaginal environment through the metabolic process of glycolysis [59, 60]. These bacteria produce lactic acid (LA), which acidifies the FRT to a low pH (pH ≤ 4.5) in women with a *Lactobacillus*‐dominant VMB, unlike the higher pH and paucity of lactobacilli found in the FRT of other mammals [63, 64] or most cellular and tissue compartments of the human body [65]. These distinctive features of the human vagina make it difficult to identify or develop an animal model that can accurately recapitulate the unique characteristics of the human VMB [66, 67].

## 4 The Composition of an Optimal and non‐Optimal VMB

**FIGURE 2:**
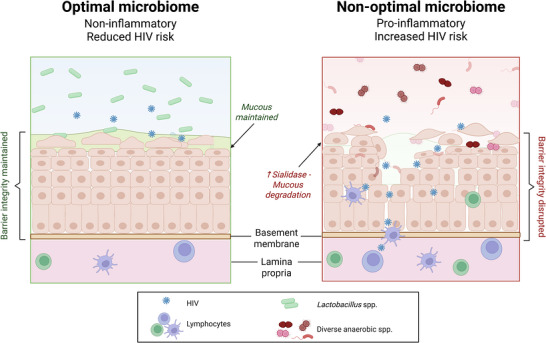
The vaginal microbiome modulates HIV susceptibility. An optimal microbiome dominated by *Lactobacillus* spp. is non‐inflammatory and reduces HIV risk, whereas a non‐optimal microbiome dominated by diverse anaerobic bacterial species is pro‐inflammatory leading to disruption of the epithelial barrier and recruitment of HIV target cells in the lamina propria. Cervicovaginal mucous is degraded by enzymes of anaerobic bacteria such as sialidases. HIV, human immunodeficiency virus. Created with BioRender.com.

Consensus in the field is transitioning from notions of ‘normal’ and ‘abnormal’ or dysbiotic VMB, with a greater focus on shifting the VMB from ‘non‐optimal’ to ‘optimal’ to improve the SRH of women [12]. There is significant variation in the VMB between individuals, ethnic groups, and geographical regions [11, 12, 68, 69, 70, 71]. Regardless of these factors, an optimal VMB, typically defined as being dominated by *L. crispatus*, is associated with favourable SRH outcomes including a decreased risk in pregnancy complications and STI acquisition (Figure 2) [12, 59, 70, 72].

A ground‐breaking study published in 2011 characterized bacterial taxa of the VMB of nearly 400 North American women was the first to define microbial clusters into community state types (CSTs) [73]. Of the five CSTs, four had a dominant *Lactobacillus* sp., with CST I, II, III and V dominated *by L. crispatus, L. gasseri, L. iners* and *L. jensenii*, respectively (Table 1). In the lower FRT, *Lactobacillus* spp. kill or suppress the growth of pathogens and non‐optimal vaginal bacteria through the production of antimicrobial and immunomodulatory factors [40, 63, 74, 75, 76]. *L. crispatus‐*dominated microbiota, which maintains the lowest vaginal pH [73, 77], is the most impervious to disruption and CST transition [12, 78]. CST III is dominated by *L. iners* and despite being frequently present in the vaginal environment, is generally considered less optimal (sub‐optimal) compared to *L. crispatus* [79, 80]. This is due in part to rapid transition of an *L. iners* dominated VMB to a non‐optimal vaginal microbiota following sexual activity and menses [81].

**TABLE 1:** Vaginal microbiota community state types (CSTs) and average vaginal pH.

CST^a^	Dominant species	pH^b^
CST I	*Lactobacillus crispatus*	4.0 ± 0.3
CST II	*Lactobacillus gasseri*	5.0 ± 0.7
CST III	*Lactobacillus iners*	4.4 ± 0.6
CST IVA	*Gardnerella vaginalis* and *Candidatus* Lachnocurva vaginae [96]	5.3 ± 0.6
CST IVB	*Gardnerella vaginalis* and *Fannyhessea vaginae*	
CST IVC	Diverse polymicrobial anaerobes, lacking *Lactobacillus* spp.	
CST V	*Lactobacillus jensenii*	4.7 ± 0.4

^a^ Community state types (CST) as defined by Ravel et al. [73], France et al. [82] and McKinnon et al. [12].

^b^ Average vaginal pH ± median absolute deviation.

The non‐optimal VMB, also defined as CST IV, is dominated by diverse or non‐*Lactobacillus* bacteria [73]. In the United States, *Lactobacillus*‐dominated CST I, II, III and V were found in 89.2% of white women, but only approximately 60% of Hispanic and black women [73]. Studies of larger data sets have allowed for further resolution of CSTs into subgroups [82], including the division of CST IV into subgroups IVA, IVB and IVC. CST IVA is dominated by *Candidatus Lachnocurva vaginae* (formerly BVAB1) with a moderate relative abundance of *Gardnerella vaginalis* and *Fannyhessea vaginae* (formerly *Atopobium vaginae*) [82, 83], IVB is dominated by *G. vaginalis* with a moderate relative abundance of *Fannyhessea vaginae*, and IVC comprises diverse polymicrobial anaerobes and a paucity in the relative abundance of *Lactobacillus spp*. [12, 73, 82, 84]. As sequencing methods have improved with shifts from 16S rRNA gene sequencing to whole genome sequencing, resolution has increased, and previously unknown bacterial species and strains have been discovered to reside in the FRT [73, 82, 85, 86]. This includes 786 prokaryotic species characterized in the FRT [87], with at least 46 species uniquely identified in African American and African women to date [85]. Studies of the multi‐kingdom composition of the VMB have additionally characterized 11 fungal species, and 4263 viral taxa [87].

The composition of the VMB is influenced by numerous factors, including endogenous and exogenous female sex hormones such as oestrogen, progesterone and hormonal contraceptives [88, 89]. VMB colonisation is altered across the menstrual and reproductive cycle, as periods of higher oestrogen in the proliferative phase are associated with increased glycogen production and deposition in the vaginal environment, a key facilitator of *Lactobacillus* colonisation [58, 90, 91, 92]. Lower oestradiol levels, such as in post‐menopausal women, are closely associated with colonisation of a diverse VMB [93, 94]. A drop in oestrogen levels just prior to menarche may present a window of increased vulnerability to HIV infection, mediated by decreased glycogen deposition and a sub‐dominance of *L. crispatus* [95, 96].

## 5 Sexual and Reproductive Health Risks of a non‐Optimal Microbiota are not Limited to Symptomatic Bacterial Vaginosis

Bacterial vaginosis (BV) is a common vaginal condition characterised by an overgrowth of diverse anaerobic bacteria, which may present clinically with symptoms of watery vaginal discharge, irritation and odour [12, 97]. BV is associated with genital inflammation and proinflammatory cytokines and an increased risk of negative SRH outcomes, including acquisition of HIV and other STIs, increased risk of pre‐term birth and low birth weight of infants, postpartum endomyometritis, and pelvic inflammatory disease [12, 98, 99, 100, 101, 102, 103]. Low birth weight is a factor in around 80% of early infant deaths and can have lifelong consequences for childhood development and chronic illness [98, 101]. Similar to women with clinical BV, asymptomatic women colonised with a non‐optimal VMB have elevated levels of proinflammatory cytokines, increased epithelial shedding and are at risk of negative health outcomes including increased HIV and STI acquisition risk [12, 104]. A non‐optimal VMB can be detected using molecular methods (e.g., 16S rRNA gene sequencing) [12], but standard clinical practice does not currently involve screening or treatment of asymptomatic patients [12, 105].

The penile microbiome of sexual partners is hypothesised to influence the composition of the VMB. BV‐associated bacteria are sexually transmissible, with a study in young Kenyan women reporting an increase in BV rates following sexual debut [106], and others finding that women with regular sexual partners experience as high as an 80% rate of BV recurrence 16 weeks post‐treatment [107, 108, 109]. Women who were administered a first‐line BV treatment regimen had a significant decrease in BV recurrence when their male partners were simultaneously treated with daily oral metronidazole and twice daily topical clindamycin cream for seven days (63% vs. 35% recurrence within 12 weeks) [110]. However, successful metronidazole treatment of women with BV and clearance of non‐optimal vaginal bacteria typically leads to the persistence of sub‐optimal *L. iners*, which can readily transition to non‐optimal VMB and BV recurrence [11, 111]. There remains a need to improve clinical practice for BV treatment as well as identifying asymptomatic women with non‐optimal VMB who are also at risk of adverse SRH outcomes.

Data from in vitro models suggest that *L. crispatus* actively maintain homeostasis by suppressing inflammation by toll‐like receptor (TLR) agonist activation [112], which may be mediated by *Lactobacillus* spp. [113, 114] or their metabolites [115]. Conversely, BV‐associated bacteria and/or their metabolites activate inflammatory responses (Figure 2) [116, 117, 118]. Temporal data suggest that a loss in relative abundance of *Lactobacillus* precedes an increase in BV‐associated bacterial colonisation by as long as two weeks. BV‐associated bacteria produce biogenic polyamines, such as putrescine and cadaverine, which increase vaginal pH and affect *Lactobacillus* spp. growth [58, 119, 120]. The BV‐associated bacteria, *G. vaginalis*, elicits inflammatory pathway activation though production of extracellular vesicles [121] and glycan degradation by sialidases [122, 123]. Loss of lactobacilli has been reported to be mediated by *Lactobacillus‐*specific bacteriophages, which increase in abundance prior to the onset of BV [123]. While the relative importance of loss of protective *Lactobacillus* spp. compared to the introduction of BV‐associated bacterial species in transition to BV is unclear, there is evidence of an interplay between the two.

## 6 Non‐Optimal Microbiota Colonisation is Associated With Increased HIV Acquisition Risk

In young South African black women, colonisation with a non‐optimal VMB is associated with a 4.4‐fold increased risk of HIV acquisition relative to women with an *L. crispatus*‐dominant VMB [11]. A second study reported a 7.19‐fold increased HIV risk, and a higher proportion of non‐optimal (diverse and non‐*Lactobacillus* spp. dominated) VMBs observed in the youngest cohort, aged 18–19 [124]. This difference in microbiome composition correlates with the disproportionately high HIV acquisition risk observed for young women in sub‐Saharan Africa [124]. It is estimated that 29% of US women of reproductive age have BV, with a similar global average prevalence (estimates range from 5%–50%), dependent on factors including ethnicity, with Black women more likely to demonstrate BV, regardless of geographical region [70, 73, 125]. Young Black South African women have markedly low rates of *L. crispatus*, with less than 50% having a *Lactobacillus*‐dominated VMB, and just 10%–20% colonized with an *L. crispatus*‐dominant VMB [11, 124, 126]. Despite a lower prevalence in this region, a *Lactobacillus*‐dominant VMB has consistently been associated with improved health outcomes and reduced HIV risk in sub‐Saharan Africa [127, 128, 129].

HIV‐1 demonstrates increased mobility in CVF from women with non‐optimal and *L. iners* dominated vaginal microbiota, compared to CVF from women with *L. crispatus*, suggesting a loss of the protective function of the mucus barrier in women with nonoptimal VMB [19]. Increased risk of HIV acquisition may also be mediated by a diverse VMB altering the vaginal proteome, increasing pro‐inflammatory cytokine production and disruption of the mucosal barrier [130, 131, 132]. However, our understanding of the importance of functional classification of VMB rather than species identity alone is still developing [58, 131, 132, 133]. While *L. iners* is typically considered suboptimal, in some women, dominance by *L. iners* in the FRT may not contribute to adverse SRH outcomes [80, 134], which may be due to the high genetic heterogeneity among *L. iners* isolates [134]. Recent studies have highlighted that microbial function may be the most important factor when defining disease states [58, 134, 135, 136]. Significant variations in the inflammatory properties and LA production were identified between vaginal *Lactobacillus* isolates of the same species from women with optimal and non‐optimal microbiota, suggesting that microbial function may be a more impactful measure compared to species identification [135].

## 7 Effector Metabolites of the VMB

Bacteria in the microbiome produce diverse metabolites with effector functions that differ between species and by microbial niche [59, 137, 138, 139]. Short‐chain fatty acids (SCFAs) are microbial metabolites that are associated with important benefits in the gut by increasing intestinal barrier integrity and reducing pro‐inflammatory cytokine production [139, 140]. Conversely, in the vaginal environment, SCFAs such as acetic acid, propionic acid, butyric acid, as well as succinic acid are produced by non‐optimal microbiota but their impact on the FRT remains to be fully defined [118]. While their impact on modulating FRT epithelial integrity has been under‐investigated to date, SCFAs can decrease barrier integrity, adhesion protein expression and initiate an inflammatory response in vaginal epithelial cells [118, 141].

Vaginal *Lactobacillus* spp. produce microbial metabolites that have been associated with positive health outcomes [142]. Hydrogen peroxide production was initially thought to be responsible for the antimicrobial properties of lactobacilli, although this is now considered implausible due to the hypoxic in vivo environment of the lower FRT and presence of CVF which inactivates H_2_O_2_ at levels necessary to elicit bactericidal activity [76, 142, 143, 144]. *Lactobacillus* spp. also produce other antimicrobial compounds including bacteriocins, surfactants and antimicrobial peptides [142, 144]. However, increasing evidence suggests that LA is a key mediator of *Lactobacillus* spp. effector functions in the lower FRT [63].

## 8 LA is a Major Effector Metabolite of Vaginal Lactobacilli

LA is an alpha‐hydroxy acid with an acid dissociation constant (pK_a_) of 3.86, dissociating into a lactate and hydrogen ion molecule with increasing pH [59]. In women with a *Lactobacillus*‐dominated VMB, the physiological range of total LA, consisting of protonated LA and lactate (non‐protonated, LA^−^), ranges from 1% ± 0.2% w/v, and at a pH of 3.5 ± 0.2, results in an approximate active (protonated) LA concentration of 0.69% w/v (76 mM) [145]. LA is produced by optimal lactobacilli at high concentrations in the hypoxic conditions of the cervicovaginal environment [142, 143, 145]. Most LA present in the lower FRT is of bacterial origin, with only 15% produced by vaginal epithelial cells [63, 146]. LA is the main acidifier in the lower FRT, and acidification of the vaginal environment through the production of LA appears to be a critical and conserved function of the lower FRT microbiome [59, 73, 78, 145]. LA has a strong buffering capacity and is more capable of maintaining a low vaginal pH compared to other organic acids present in the FRT, including SCFAs, due to LA having a lower pKa [75, 118, 145].

LA is a chiral molecule that exists as two stereoisomers, D‐LA and L‐LA [77]. *L. crispatus* and *L. gasseri* produce both isomers, *L. jensenii* produces only D‐LA, and *L. iners* is the only common species that produces L‐ but not D‐LA [60]. The lack of D‐LA production by *L. iners* is of interest given the less favourable SRH outcomes associated with an *L. iners*‐dominated VMB [11, 33, 79, 147]. D‐LA is associated with more potent blocking of *Chlamydia trachomatis* infection of FRT epithelial cells [33], while L‐LA elicits more potent inactivation of HIV compared to D‐LA (17‐fold) at threshold concentration (0.3%) [75, 148]. However, some strains of *L. iners* produce less LA overall compared to *L. crispatus* and are therefore less efficient at acidifying the vaginal environment, which may bet explain the differences in health outcomes [63, 81, 149] although strain level variations may also play a role [134].

## 9 The Potential Role of LA in HIV Prevention

LA has been identified in vitro to have protective effects in the FRT beyond acidification of the vaginal environment [59, 63, 76]. LA (which is protonated), but not lactate, has potent virucidal activity against HIV [75, 148] and is anticipated to inactivate both cell‐free and cell‐associated HIV [19, 148, 150, 151]. LA also has diverse antimicrobial effects against a range of BV‐associated bacteria and STI pathogens [59] including *Neisseria gonorrhoeae*, *Chlamydia trachomatis* and *Escherichia coli* [143, 152, 153]. Our pilot data indicates that LA can kill *L. iners* but not *L. crispatus* in vitro [154], suggesting a strategy to promote colonisation with *L. crispatus* in vivo.

LA has immunomodulatory and anti‐inflammatory properties on cervicovaginal epithelial cells in vitro, producing increased concentrations of the anti‐inflammatory cytokine IL‐1RA and preventing the increase of pro‐inflammatory cytokines such as IL‐6, TNF‐α and IL‐8 after TLR agonist stimulation [115]. These findings suggest that LA may decrease inflammation in the FRT, with consequences for immune cell activation and recruitment. In addition, LA enhances epithelial barrier integrity in vitro by increasing expression of tight junction proteins, which may be a mechanism for reducing HIV paracellular translocation within the FRT (Figure 1A) [141, 155, 156]. Emerging evidence suggests that LA may also modulate HIV translocation mediated by internalisation and transcytosis through an intact FRT epithelial barrier (Figure 1B) [156], although these findings require confirmation using primary FRT tissues.

## 10 Modulating the VMB to Prevent HIV and STI Transmission

Given the integral role of the VMB in influencing HIV and STI transmission, modulation of the VMB offers the potential to enhance protection from these pathogens. Therapeutic modulation of the VMB is typically undertaken only for the treatment of symptomatic BV, where the standard of care is antibiotic therapy (e.g., metronidazole) [105]. However, there are significant issues with frequent BV recurrence of approximately 60% within 6 months following treatment of the affected woman [157, 158]. Rates of recurrence can increase with each subsequent BV diagnosis [106] if the male partner is not concurrently treated with antibiotics [110]. This may be due to lack of clearance of BV‐associated bacteria by the antibiotic and/or persistence of *L. iners*, which readily transitions to a non‐optimal VMB (i.e., BV) within 12 weeks of treatment [58, 78, 159]. Antibiotic use may result in side‐effects, off‐target effects on other commensal bacteria, and contribute to antibiotic resistance [160, 161]. There is a clear need for the development of more effective antibiotic‐sparing treatments that can overcome the recurrence of BV. These include the potential for live biotherapeutic products which administer *L. crispatus* strains to promote an optimal VMB [162, 163, 164, 165], and/or the identification of specific metabolites produced by the optimal VMB that can be applied for therapeutic purposes to support *L. crispatus* colonisation [154, 166].

Novel approaches for efficacious, persistent BV cure are under investigation and may be exploited to protect against HIV and STI infection. Key considerations for viable treatment options include generalisability for a wide range of women, cost and accessibility, pathways towards regulatory approval, and acceptability to end users. Prebiotic strategies involve providing nutrients which are selectively utilized by optimal bacterial species, such as increasing glycogen availability with oestrogen treatment, or restricting nutrients necessary for growth of non‐optimal species [96, 167, 168]. Probiotics that are purported to alter the VMB composition are readily available over‐the‐counter, generally as repurposed oral dietary supplements containing *Lactobacillus* strains native to the gut and not typically found in the FRT. While these strains may modulate the VMB due to similarity with the gut microbiome, their efficacy for achieving an optimal VMB is unclear [169, 170, 171]. Efficacy may be improved using intravaginally administered live bacteria derived from optimal VMB such as *L. crispatus* [162, 163, 164, 165, 172]. A placebo‐controlled, double‐blind phase IIb trial of a live biotherapeutic product of *L. crispatus* known as LACTIN‐V saw modest reductions in BV recurrence at 12 weeks with 45% in the placebo group compared to 30% in the LACTIN‐V intervention group [164]. Further, LACTIN‐V was demonstrated to be safe and tolerable for use in pregnancy [173], a crucial consideration for modifying the VMB to reduce risk of spontaneous pre‐term birth and safe use in women with potential for pregnancy. LACTIN‐V was also shown to establish transient colonisation in a cohort of Black South African women, demonstrating potential feasibility for an optimised live biotherapeutic strategy in this population [172]. Using a consortium of several *L. crispatus* strains isolated from women with optimal VMB may improve the potential for colonisation compared to a single strain [163, 165], although bringing this approach to market as an approved therapeutic through regulatory pathways may be more challenging [58].

## 11 Opportunities for Metabolite‐Based Therapeutics in HIV Prevention

Mounting evidence suggests that LA is important in establishing and maintaining an optimal VMB, by maintaining a low vaginal pH and promoting the growth of *Lactobacillus* spp. while inhibiting non‐*Lactobacillus* spp. [63]. If LA is demonstrated to be an effective therapeutic for altering the VMB, it could hypothetically be used in combination with other interventions such as topical PrEP and hormonal contraceptives in multi‐purpose prevention technologies such as gels, films or IVRs to deliver positive SRH outcomes for women in low‐income settings [174].

There is an unmet need for high‐quality clinical evidence to support the use of LA‐containing products for BV cure or VMB modulation. A 2021 systematic review determined that the few existing studies had limitations and conflicting findings regarding the efficacy of LA‐containing products compared to metronidazole or a placebo [166]. Of assessed studies, three trials compared the efficacy of an LA‐containing product (e.g., gel, pessary or douching product) to metronidazole for BV cure [175, 176, 177]. One found LA to be equivalent to metronidazole [175], while two studies found LA to be significantly inferior [176, 177]. Two other studies included a control group receiving a placebo or no treatment [178, 179]. One reported LA to be superior to no treatment [178] and the other found LA to be equivalent to placebo [179]. One study did not have a placebo or untreated control group, and compared groups treated with two LA‐containing products after metronidazole treatment of BV [180]. In these studies, LA‐containing products did not significantly impact the vaginal microbiota composition as measured by 16S rRNA gene sequencing. However, adequately powered and rigorous randomised trials with accompanying vaginal microbiota data are needed to evaluate the efficacy of LA as a BV treatment strategy [166]. The VITA study assessed a topical LA gel compared to oral metronidazole for treatment of BV and found that the LA gel was more acceptable and tolerable than antibiotic treatment for BV [161, 181]. Although metronidazole led to short‐term BV resolution in 70% of participants and LA gels led to resolution in only 47% of women, both groups experienced a 70% rate of recurrence of symptoms within six months, and LA gel usage was associated with fewer reported side effects [182]. Another recent trial of a 4.5% LA and glycogen‐containing gel used twice weekly for eight weeks found that asymptomatic participants experienced a significant reduction in vaginal pH and Nugent scores compared to baseline [183]. As these studies lacked a placebo arm, more rigorous and placebo‐controlled trials are still required to determine effects specific to LA‐containing gels [182, 183].

LA‐containing products currently available over‐the‐counter have unfavourable properties including high osmolality, are associated with high cytotoxicity and epithelial barrier disruption and/or elicit pro‐inflammatory cytokine responses in a validated 3D organotypic vaginal in vitro model [179, 184]. Pre‐clinical evaluation of safety for vaginal products is challenging, with models such as the rabbit vaginal irritation test which are required for regulatory approval in some jurisdictions having limited human relevancy. In vitro models are currently under investigation as alternatives for assessing and predicting vaginal irritation of intravaginal products [185]. Important considerations in the development of a safe and effective LA‐containing product include maintaining a low pH, LA concentration, D‐ and L‐ isomer composition, ease of use such as sustained‐release vaginal delivery, gel osmolality and the requirement for non‐toxic additional ingredients including preservatives required to maintain shelf life [166, 184, 186].

If LA is demonstrated to modulate the VMB of women in vivo, it could be used as part of several adjunct approaches to help reduce HIV acquisition in women with non‐optimal VMB. For example, LA could be used in combination with antibiotic treatment to maximize ‘weeding’ non‐optimal BV‐associated bacteria as well as *L. iners* followed by ‘seeding’ with an *L. crispatus*‐based live biotherapeutic product to promote recolonisation by optimal vaginal *Lactobacillus* spp. LA could be used periodically to promote live biotherapeutic product colonisation following microbiome disruption events such as menses or sexual activity [78] or incorporated as an ingredient in sustained delivery vaginal formulations for SRH interventions to help maintain an optimal vaginal microbiota and mucosal environment. Novel treatment strategies are required to reduce the substantial risks associated with BV, including increased likelihood of HIV and STI acquisition and spontaneous pre‐term birth. Modulating the FRT towards an optimal microbial composition, low pH and increased LA concentration may have positive effects for HIV prevention and SRH more broadly.

## Funding

This project was supported by Project Grant (GNT1164982) awarded to GT and ACH from the National Health and Medical Research Council (NHMRC) of Australia and NHMRC Senior Research Fellowship GNT1117748 to GT. BJ was supported by the Commonwealth through an Australian Government Research Training Program Scholarship from Monash University [DOI: https://doi.org/10.82133/C42F‐K220]. We gratefully acknowledge the contribution to this work of the Victorian Operational Infrastructure Support Program received by the Burnet Institute.

## Conflicts of Interest

GT and ACH are coinventors on a granted US patent on the immunomodulatory effects of lactic acid on cervicovaginal epithelial cells. GT is also an inventor on a submitted patent application on a novel vaginal lactic acid gel formulation. The other authors have declared that no competing interests exist.

## Data Availability

Data sharing not applicable to this article as no datasets were generated or analysed during the current study

## References

[1] UNAIDS AIDS, Crisis and the Power to Transform: UNAIDS Global AIDS Update 2025 (Joint United Nations Program on HIV/AIDS, 2025).

[2] M. C. Boily , R. F. Baggaley , L. Wang , et al., “Heterosexual Risk of HIV‐1 Infection per Sexual Act: Systematic Review and Meta‐Analysis of Observational Studies,” Lancet Infectious Diseases 9 (2009): 118–129. 19179227 10.1016/S1473-3099(09)70021-0 PMC4467783

[3] J. B. Griffin , K. Ridgeway , E. Montgomery , et al., “Vaginal Ring Acceptability and Related Preferences Among Women in Low‐ and Middle‐Income Countries: A Systematic Review and Narrative Synthesis,” PLoS ONE 14 (2019): e0224898. 31703094 10.1371/journal.pone.0224898 PMC6839883

[4] M. K. Shapley‐Quinn , S. Tenza , D. Jensen , et al., “Adolescent Girls and Young Women Overcoming Adherence Challenges With Vaginal and Oral PrEP Use: A Longitudinal Qualitative Study From a Crossover Trial in South Africa, Uganda, and Zimbabwe,” Aids and Behavior 28 (2024): 4209–4223. 39343865 10.1007/s10461-024-04503-y PMC11586305

[5] M. Atujuna , K. Williams , S. T. Roberts , et al., “We Choose: Adolescent Girls and Young Women's Choice for an HIV Prevention Product in a Cross‐Over Randomized Clinical Trial Conducted in South Africa, Uganda, and Zimbabwe,” PLoS ONE 19 (2024): e0308577. 39208281 10.1371/journal.pone.0308577 PMC11361692

[6] E. Sanchez Armengol , F. Veider , G. Millotti , G. Kali , A. Bernkop‐Schnürch , and F. Laffleur , “Exploring the Potential of Vaginal Drug Delivery: Innovations, Efficacy, and Therapeutic Prospects,” Journal of Pharmacy and Pharmacology 77 (2025): 1149–1165. 40569139 10.1093/jpp/rgaf045

[7] S. K. Patel , H. Agashe , D. L. Patton , et al., “Tenofovir Vaginal Film as a Potential MPT Product Against HIV‐1 and HSV‐2 Acquisition: Formulation Development and Preclinical Assessment in Non‐Human Primates,” Frontiers Reproductive Health 5 (2023): 1217835. 10.3389/frph.2023.1217835 PMC10449455 37638127

[8] A. Hemmerling , S. Moore , and K. Yang , “Expanding the Pipeline for Multipurpose Prevention Technologies: Compounds With Potential Activity to Prevent or Treat HIV and Other STIs,” Sexually Transmitted Infections 99 (2023): 203–207. 36878691 10.1136/sextrans-2022-055647

[9] J. Li , S. K. Patel , Y. Sweeney , et al., “Engineering Vaginal Film Platform for Mucoadhesion and Sustained Drug Release for HIV‐1 Prevention,” Journal of Controlled Release: Official Journal of the Controlled Release Society 379 (2025): 696–707.39814320 10.1016/j.jconrel.2025.01.011

[10] X. Tong , S. K. Patel , J. Li , et al., “Development and Evaluation of Nanoparticles‐in‐Film Technology to Achieve Extended In Vivo Exposure of MK‐2048 for HIV Prevention,” Polymers (Basel) 14 (2022): 1196. 35335526 10.3390/polym14061196 PMC8955144

[11] C. Gosmann , M. N. Anahtar , S. A. Handley , et al., “Lactobacillus‐Deficient Cervicovaginal Bacterial Communities Are Associated With Increased HIV Acquisition in Young,” South African Women Immunity 46 (2017): 29–37. 28087240 10.1016/j.immuni.2016.12.013 PMC5270628

[12] L. R. McKinnon , S. L. Achilles , C. S. Bradshaw , et al., “The Evolving Facets of Bacterial Baginosis: Implications for HIV Transmission,” Aids Research and Human Retroviruses 35 (2019): 219–228. 30638028 10.1089/aid.2018.0304 PMC6434601

[13] B. Zhu , Z. Tao , L. Edupuganti , M. G. Serrano , and G. A. Buck , “Roles of the Microbiota of the Female Reproductive Tract in Gynecological and Reproductive Health,” Microbiology and Molecular Biology Reviews 86, no. 4 (2022): e00181211. 10.1128/mmbr.00181-21 PMC9769908 36222685

[14] A. M. Carias , S. McCoombe , M. McRaven , et al., “Defining the Interaction of HIV‐1 With the Mucosal Barriers of the Female Reproductive Tract,” Journal of Virology 87 (2013): 11388–11400. 23966398 10.1128/JVI.01377-13 PMC3807311

[15] D. J. Stieh , D. Maric , Z. L. Kelley , et al., “Vaginal Challenge With an SIV‐based Dual Reporter System Reveals That Infection Can Occur Throughout the Upper and Lower Female Reproductive Tract,” Plos Pathogens 10 (2014): e1004440. 25299616 10.1371/journal.ppat.1004440 PMC4192600

[16] A. M. Carias and T. J. Hope , “Barriers of Mucosal Entry of HIV/SIV,” Current Immunology Reviews 15 (2019): 4–13. 31853241 10.2174/1573395514666180604084404 PMC6919325

[17] F. Taherali , F. Varum , and A. W. Basit , “A Slippery Slope: On the Origin, Role and Physiology of Mucus,” Advanced Drug Delivery Reviews 124 (2018): 16–33. 29108861 10.1016/j.addr.2017.10.014

[18] M. W. Y. Choi , C. A. Isidoro , and A. Gillgrass , “Mechanisms of Mucosal Immunity at the Female Reproductive Tract Involved in Defense Against HIV Infection,” Current Opinion in Virology 66 (2024): 101398. 38484474 10.1016/j.coviro.2024.101398

[19] T. Hoang , E. Toler , K. DeLong , et al., “The Cervicovaginal Mucus Barrier to HIV‐1 Is Diminished in Bacterial Vaginosis,” Plos Pathogens 16 (2020): e1008236. 31971984 10.1371/journal.ppat.1008236 PMC6999914

[20] L. Monin , E. M. Whettlock , and V. Male , “Immune Responses in the Human Female Reproductive Tract,” Immunology 160 (2020): 106–115. 31630394 10.1111/imm.13136 PMC7218661

[21] O. Gutzeit , A. Gulati , Z. Izadifar , et al., “Cervical Mucus in Linked Human Cervix and Vagina Chips Modulates Vaginal Dysbiosis,” NPJ Womens Health 3 (2025): 5. 39896100 10.1038/s44294-025-00054-2 PMC11779628

[22] A. Nazli , O. Chan , W. N. Dobson‐Belaire , et al., “Exposure to HIV‐1 Directly Impairs Mucosal Epithelial Barrier Integrity Allowing Microbial Translocation,” Plos Pathogens 6 (2010): e1000852. 20386714 10.1371/journal.ppat.1000852 PMC2851733

[23] M. Girard , J. Mahoney , Q. Wei , et al., “Genital Infection of Female Chimpanzees With Human Immunodeficiency Virus Type 1,” Aids Research and Human Retroviruses 14 (1998): 1357–1367. 9788677 10.1089/aid.1998.14.1357

[24] V. Caputo , M. Libera , S. Sisti , B. Giuliani , and R. A. Diotti , “The Initial Interplay Between HIV and Mucosal Innate Immunity,” Frontiers in Immunology 14 (2023): 1104423. 36798134 10.3389/fimmu.2023.1104423 PMC9927018

[25] A. A. Bhat , S. Uppada , I. W. Achkar , et al., “Tight Junction Proteins and Signaling Pathways in Cancer and Inflammation: A Functional Crosstalk,” Frontiers in Physiology 9 (2019): 1942. 30728783 10.3389/fphys.2018.01942 PMC6351700

[26] C. Cunniffe , F. Ryan , H. Lambkin , and B. Brankin , “Expression of Tight and Adherens Junction Proteins in Cervical Neoplasia,” British Journal of Biomedical Science 69 (2012): 147–153. 23304789

[27] C. Nold , L. Anton , A. Brown , and M. Elovitz , “Inflammation Promotes a Cytokine Response and Disrupts the Cervical Epithelial Barrier: A Possible Mechanism of Premature Cervical Remodeling and Preterm Birth,” American Journal of Obstetrics and Gynecology 206 (2012): 208.e1–208.e2087. 10.1016/j.ajog.2011.12.036 22285171

[28] A. R. Thurman , N. Yousefieh , N. Chandra , et al., “Comparison of Mucosal Markers of Human Immunodeficiency Virus Susceptibility in Healthy Premenopausal Versus Postmenopausal Women,” Aids Research and Human Retroviruses 33 (2017): 807–819. 28398069 10.1089/aid.2016.0320 PMC5564035

[29] R. J. Shattock and J. P. Moore , “Inhibiting Sexual Transmission of HIV‐1 Infection,” Nature Reviews Microbiology 1 (2003): 25–34. 15040177 10.1038/nrmicro729

[30] A. Mohammadi , S. Bagherichimeh , Y. Choi , et al., “Immune Parameters of HIV Susceptibility in the Female Genital Tract Before and After Penile‐Vaginal Sex,” Communications Medicine 2 (2022): 60. 35637661 10.1038/s43856-022-00122-7 PMC9142516

[31] K. B. Arnold , A. Burgener , K. Birse , et al., “Increased Levels of Inflammatory Cytokines in the Female Reproductive Tract Are Associated With Altered Expression of Proteases, Mucosal Barrier Proteins, and an Influx of HIV‐Susceptible Target Cells,” Mucosal Immunology 9 (2016): 194–205. 26104913 10.1038/mi.2015.51

[32] K. A. Powers , C. Poole , A. E. Pettifor , and M. S. Cohen , “Rethinking the Heterosexual Infectivity of HIV‐1: A Systematic Review and Meta‐Analysis,” Lancet Infectious Diseases 8 (2008): 553–563. 18684670 10.1016/S1473-3099(08)70156-7 PMC2744983

[33] V. L. Edwards , S. B. Smith , E. J. McComb , et al., “The Cervicovaginal Microbiota‐Host Interaction Modulates Chlamydia Trachomatis Infection,” MBio 10 (2019): e01548–19. 10.1128/mBio.01548-19 PMC6692509 31409678

[34] A. S. Hinderfeld , N. Phukan , A.‐K. Bär , A. M. Roberton , and A. Simoes‐Barbosa , “Cooperative Interactions Between Trichomonas vaginalis and Associated Bacteria Enhance Paracellular Permeability of the Cervicovaginal Epithelium by Dysregulating Tight Junctions,” Infection and Immunity 87 (2019): e00141–19. 10.1128/IAI.00141-19 PMC6479042 30858343

[35] E. Armstrong and R. Kaul , “Beyond Bacterial Vaginosis: Vaginal Lactobacilli and HIV Risk,” Microbiome 9 (2021): 239. 34893070 10.1186/s40168-021-01183-x PMC8665571

[36] P. Y. Uc , J. Miranda , A. Raya‐Sandino , et al., “E7 Oncoprotein From Human Papillomavirus 16 Alters Claudins Expression and the Sealing of Epithelial Tight Junctions,” International Journal of Oncology 57 (2020): 905–924. 32945372 10.3892/ijo.2020.5105 PMC7473757

[37] K. L. Nunn , Y.‐Y. Wang , D. Harit , et al., “Enhanced Trapping of HIV‐1 by Human Cervicovaginal Mucus Is Associated With Lactobacillus Crispatus‐Dominant Microbiota,” MBio 6 (2015): e01084–15. 10.1128/mBio.01084-15 PMC4611035 26443453

[38] V. Barreto‐de‐Souza , A. Arakelyan , L. Margolis , and C. Vanpouille , “HIV‐1 Vaginal Transmission: Cell‐Free or Cell‐Associated Virus?,” American Journal of Reproductive Immunology 71 (2014): 589–599. 24730358 10.1111/aji.12240 PMC4517939

[39] M. Ghosh , J. V. Fahey , Z. Shen , et al., “Anti‐HIV Activity in Cervical‐Vaginal Secretions From HIV‐Positive and ‐Negative Women Correlate With Innate Antimicrobial Levels and IgG Antibodies,” PLoS ONE 5 (2010): e11366. 20614007 10.1371/journal.pone.0011366 PMC2894072

[40] D. Tyssen , Y.‐Y. Wang , J. A. Hayward , et al., “Anti‐HIV‐1 Activity of Lactic Acid in Human Cervicovaginal Fluid,” mSphere 3 (2018): e00055–18. 10.1128/mSphere.00055-18 PMC6034077 29976641

[41] P. B. Pendergrass , M. W. Belovicz , and C. A. Reeves , “Surface Area of the Human Vagina as Measured From Vinyl Polysiloxane Casts,” Gynecologic and Obstetric Investigation 55 (2003): 110–113. 12771458 10.1159/000070184

[42] F. Hladik and T. J. Hope , “HIV Infection of the Genital Mucosa in Women,” Current HIV/AIDS Reports 6 (2009): 20–28. 19149993 10.1007/s11904-009-0004-1

[43] S. K. Lai , K. Hida , S. Shukair , et al., “Human Immunodeficiency Virus Type 1 Is Trapped by Acidic but Not by Neutralized human Cervicovaginal Mucus,” Journal of Virology 83 (2009): 11196–11200. 19692470 10.1128/JVI.01899-08 PMC2772788

[44] J. Jewanraj , S. Ngcapu , and L. J. P. Liebenberg , “Semen: A Modulator of Female Genital Tract Inflammation and a Vector for HIV‐1 Transmission,” American Journal of Reproductive Immunology 86 (2021): e13478. 34077596 10.1111/aji.13478 PMC9286343

[45] D. Sodora , A. Gettie , C. Miller , and P. Marx , “Vaginal Transmission of SIV: Assessing Infectivity and Hormonal Influences in Macaques Inoculated With Cell‐Free and Cell‐Associated Viral Stocks,” Aids Research and Human Retroviruses 14 Suppl 1 (1998): S119–23. 9581895

[46] A. Yasen , R. Herrera , K. Rosbe , K. Lien , and S. M. Tugizov , “Release of HIV‐1 Sequestered in the Vesicles of Oral and Genital Mucosal Epithelial Cells by Epithelial‐Lymphocyte Interaction,” Plos Pathogens 13 (2017): e1006247. 28241053 10.1371/journal.ppat.1006247 PMC5344537

[47] M. P. Carreno , C. Krieff , T. Irinopoulou , M. D. Kazatchkine , and L. Belec , “Enhanced Transcytosis of R5‐Tropic Human Immunodeficiency Virus Across Tight Monolayer of Polarized Human Endometrial Cells Under Pro‐Inflammatory Conditions,” Cytokine 20 (2002): 289–294. 12633571 10.1006/cyto.2002.2009

[48] A. M. Carias , M. Anderson , M. McRaven , E. Allen , A. J. Fought , and T. J. Hope , “Transcytosis as a Mechanism of HIV‐1 Entry Into Columnar Epithelial Explants of the Female Reproductive Tract,” Aids Research and Human Retroviruses 41 (2025): 167–173. 39665594 10.1089/aid.2024.0045 PMC11971551

[49] M. D. Bobardt , U. Chatterji , S. Selvarajah , et al., “Cell‐Free Human Immunodeficiency Virus Type 1 Transcytosis Through Primary Genital Epithelial Cells,” Journal of Virology 81 (2007): 395–405. 17050597 10.1128/JVI.01303-06 PMC1797244

[50] A. Yasen , R. Herrera , K. Rosbe , K. Lien , and S. M. Tugizov , “HIV Internalization Into Oral and Genital Epithelial Cells by Endocytosis and Macropinocytosis Leads to Viral Sequestration in the Vesicles,” Virology 515 (2018): 92–107. 29277006 10.1016/j.virol.2017.12.012 PMC5823522

[51] A. J. Quayle , C. Xu , K. H. Mayer , and D. J. Anderson , “T Lymphocytes and Macrophages, but Not Motile Spermatozoa, Are a Significant Source of Human Immunodeficiency Virus in Semen,” Journal of Infectious Diseases 176 (1997): 960–968. 9333154 10.1086/516541

[52] P. Lawrence , D. Portran , R. Terrasse , et al., “Selective Transmigration of Monocyte‐Associated HIV‐1 Across a Human Cervical Monolayer and Its Modulation by Seminal Plasma,” AIDS 26 (2012): 785–796. 22495223 10.1097/QAD.0b013e328351426e

[53] C. J. Chancey , K. V. Khanna , J. F. Seegers , et al., “Lactobacilli‐Expressed Single‐Chain Variable Fragment (scFv) Specific for Intercellular Adhesion Molecule 1 (ICAM‐1) Blocks Cell‐Associated HIV‐1 Transmission Across a Cervical Epithelial Monolayer,” Journal of Immunology 176 (2006): 5627–5636. 10.4049/jimmunol.176.9.5627 16622032

[54] P. Chen , W. Hübner , M. A. Spinelli , and B. K. Chen , “Predominant Mode of Human Immunodeficiency Virus Transfer Between T Cells Is Mediated by Sustained Env‐Dependent Neutralization‐Resistant Virological Synapses,” Journal of Virology 81 (2007): 12582–12595. 17728240 10.1128/JVI.00381-07 PMC2169007

[55] L. Bracq , M. Xie , S. Benichou , and J. Bouchet , “Mechanisms for Cell‐to‐Cell Transmission of HIV‐1,” Frontiers in Immunology 9 (2018): 260–260. 29515578 10.3389/fimmu.2018.00260 PMC5825902

[56] C. Huttenhower , D. Gevers , R. Knight , et al., “Structure, Function and Diversity of the Healthy Human Microbiome,” Nature 486 (2012): 207–214. 22699609 10.1038/nature11234 PMC3564958

[57] I. Rowland , G. Gibson , A. Heinken , et al., “Gut Microbiota Functions: Metabolism of Nutrients and Other Food Components,” European Journal of Nutrition 57 (2018): 1–24. 10.1007/s00394-017-1445-8 PMC5847071 28393285

[58] M. France , M. Alizadeh , S. Brown , B. Ma , and J. Ravel , “Towards a Deeper Understanding of the Vaginal Microbiota,” Nature Microbiology 7 (2022): 367–378. 10.1038/s41564-022-01083-2 PMC8910585 35246662

[59] M. Aldunate , D. Srbinovski , A. C. Hearps , et al., “Antimicrobial and Immune Modulatory Effects of Lactic Acid and Short Chain Fatty Acids Produced by Vaginal Microbiota Associated With Eubiosis and Bacterial Vaginosis,” Frontiers in Physiology 6 (2015): 164. 26082720 10.3389/fphys.2015.00164 PMC4451362

[60] S. Witkin and I. Linhares , “Why Do Lactobacilli Dominate the Human Vaginal Microbiota?,” BJOG 124 (2017): 606–611. 28224747 10.1111/1471-0528.14390

[61] P. Mirmonsef , A. L. Hotton , D. Gilbert , et al., “Free Glycogen in Vaginal Fluids Is Associated With Lactobacillus Colonization and Low Vaginal pH,” PLoS ONE 9 (2014): e102467. 25033265 10.1371/journal.pone.0102467 PMC4102502

[62] C. van der Veer , R. Y. Hertzberger , S. M. Bruisten , et al., “Comparative Genomics of Human Lactobacillus Crispatus Isolates Reveals Genes for Glycosylation and Glycogen Degradation: Implications for in Vivo Dominance of the Vaginal Microbiota,” Microbiome 7 (2019): 49. 30925932 10.1186/s40168-019-0667-9 PMC6441167

[63] G. Tachedjian , M. Aldunate , C. S. Bradshaw , and R. A. Cone , “The Role of Lactic Acid Production by Probiotic Lactobacillus Species in Vaginal Health,” Research in Microbiology 168 (2017): 782–792. 28435139 10.1016/j.resmic.2017.04.001

[64] G. J. Daggett Jr. , C. Zhao , F. Connor‐Stroud , P. Oviedo‐Moreno , et al., “Comparison of the Vaginal Environment in Rhesus and Cynomolgus Macaques Pre‐ and Post‐Lactobacillus Colonization,” Journal of Medical Primatology 46 (2017): 232–238. 28488364 10.1111/jmp.12264 PMC5597444

[65] Y. L. Zhang , J. C. Feng , L. J. Ke , et al., “Mechanisms Underlying the Regulation of Intracellular and Luminal pH in Vaginal Epithelium,” Journal of Cellular Physiology 234 (2019): 15790–15799. 30697740 10.1002/jcp.28237

[66] A. A. Wolfarth , T. M. Smith , D. VanInsberghe , et al., “A human Microbiota‐Associated Murine Model for Assessing the Impact of the Vaginal Microbiota on Pregnancy Outcomes,” Frontiers in Cellular and Infection Microbiology 10 (2020): 570025. 33123496 10.3389/fcimb.2020.570025 PMC7574503

[67] C. A. Langner , A. M. Ortiz , J. K. Flynn , H. Kendall , L. A. Lagenaur , and J. M. Brenchley , “The Vaginal Microbiome of Nonhuman Primates Can be Only Transiently Altered to Become Lactobacillus Dominant Without Reducing Inflammation,” Microbiology Spectrum 9 (2021): e0107421. 34756073 10.1128/Spectrum.01074-21 PMC8579922

[68] M. N. Anahtar , D. B. Gootenberg , C. M. Mitchell , and D. S. Kwon , “Cervicovaginal Microbiota and Reproductive Health: The Virtue of Simplicity,” Cell Host & Microbe 23 (2018): 159–168. 29447695 10.1016/j.chom.2018.01.013

[69] Y. Moosa , D. Kwon , T. de Oliveira , and E. B. Wong , “Determinants of Vaginal Microbiota Composition,” Frontiers in Cellular and Infection Microbiology 10 (2020): 467. 32984081 10.3389/fcimb.2020.00467 PMC7492712

[70] M. G. Serrano , H. I. Parikh , J. P. Brooks , et al., “Racioethnic Diversity in the Dynamics of the Vaginal Microbiome During Pregnancy,” Nature Medicine 25 (2019): 1001–1011. 10.1038/s41591-019-0465-8 PMC6746180 31142850

[71] E. Vinerbi , F. Chillotti , A. Maschio , et al., “Lactobacillus Iners Dominates the Vaginal Microbiota of Healthy Italian Women of Reproductive Age,” mSystems 10, no. 12 (2025): e0098325. 41288406 10.1128/msystems.00983-25 PMC12710342

[72] Integrative HMP (iHMP) Research Network Consortium 2019 . “The Integrative Human Microbiome Project,” Nature 569:641–648. 10.1038/s41586-019-1238-8 PMC6784865 31142853

[73] J. Ravel , P. Gajer , Z. Abdo , et al., “Vaginal Microbiome of Reproductive‐age Women,” PNAS 108 (2011): 4680–4687. 20534435 10.1073/pnas.1002611107 PMC3063603

[74] E. Miller , D. Beasley , R. Dunn , and E. Archie , “Lactobacilli Dominance and Vaginal pH: Why Is the Human Vaginal Microbiome Unique?,” Frontiers in Microbiology 7 (2016): 1936. 28008325 10.3389/fmicb.2016.01936 PMC5143676

[75] M. Aldunate , D. Tyssen , A. Johnson , et al., “Lactic Acid Produced by Optimal Vaginal Lactobacillus spp. Potently and Specifically Inactivates HIV‐1 in Vitro by Targeting the Viral RNA Genome and Reverse Transcriptase,” Plos Pathogens 21 (2025): e1013594. 41071850 10.1371/journal.ppat.1013594 PMC12527216

[76] D. E. O'Hanlon , T. R. Moench , and R. A. Cone , “In Vaginal Fluid, Bacteria Associated With Bacterial Vaginosis Can be Suppressed With Lactic Acid but Not Hydrogen Peroxide,” BMC Infectious Diseases [Electronic Resource] 11 (2011): 200. 21771337 10.1186/1471-2334-11-200 PMC3161885

[77] S. S. Witkin , H. Mendes‐Soares , I. M. Linhares , A. Jayaram , W. J. Ledger , and L. J. Forney , “Influence of Vaginal Bacteria and D‐ and L‐Lactic Acid Isomers on Vaginal Extracellular Matrix Metalloproteinase Inducer: Implications for Protection Against Upper Genital Tract Infections,” MBio 4, no. 4 (2013): e00460–13. 10.1128/mBio.00460-13 PMC3735189 23919998

[78] P. Gajer , R. M. Brotman , G. Bai , et al., “Temporal Dynamics of the Human Vaginal Microbiota,” Science Translational Medicine 4 (2012): 132ra52–132ra52. 10.1126/scitranslmed.3003605 PMC3722878 22553250

[79] M. I. Petrova , G. Reid , M. Vaneechoutte , and S. Lebeer , “Lactobacillus Iners: Friend or Foe?,” Trends in Microbiology 25 (2017): 182–191. 27914761 10.1016/j.tim.2016.11.007

[80] J. B. Holm , K. A. Carter , J. Ravel , and R. M. Brotman , “Lactobacillus Iners and Genital Health: Molecular Clues to an Enigmatic Vaginal Species,” Current Infectious Disease Reports 25 (2023): 67–75. 37234911 10.1007/s11908-023-00798-5 PMC10209668

[81] M. Vaneechoutte , “Lactobacillus Iners, the Unusual Suspect,” Research in Microbiology 168 (2017): 826–836. 28951208 10.1016/j.resmic.2017.09.003

[82] M. T. France , B. Ma , P. Gajer , et al., “VALENCIA: A Nearest Centroid Classification Method for Vaginal Microbial Communities Based on Composition,” Microbiome 8 (2020): 166–166. 33228810 10.1186/s40168-020-00934-6 PMC7684964

[83] J. B. Holm , M. T. France , B. Ma , et al., “Comparative Metagenome‐Assembled Genome Analysis of Candidatus Lachnocurva Vaginae, Formerly Known as Bacterial Vaginosis‐Associated Bacterium‐1 (BVAB1),” Frontiers in Cellular and Infection Microbiology 10 (2020): 117. 32296647 10.3389/fcimb.2020.00117 PMC7136613

[84] S. Konschuh , T. Jayaprakash , A. Dolatabadi , E. Dayo , H. Ramay , and L. Sycuro , “Reclassification of Atopobium Vaginae as Three Novel Fannyhessea Species: Implications for Understanding Their Role in Bacterial Vaginosis,” Sexually Transmitted Infections 97 (2021): A18–A18.

[85] M. Hayward , S. Bloom , M. France , et al., “Construction of a Comprehensive Bacterial Genome Catalogue for the Female Genital Tract Identifies Hundreds of New Species,” JIAS 24 (2021): 46.

[86] B. Ma , M. T. France , J. Crabtree , et al., “A Comprehensive Non‐Redundant Gene Catalog Reveals Extensive Within‐Community Intraspecies Diversity in the Human Vagina,” Nature Communications 11 (2020): 940. 10.1038/s41467-020-14677-3 PMC7044274 32103005

[87] L. Huang , R. Guo , S. Li , et al., “A Multi‐Kingdom Collection of 33,804 Reference Genomes for the Human Vaginal Microbiome,” Nature Microbiology 9 (2024): 2185–2200. 10.1038/s41564-024-01751-5 PMC11306104 38907008

[88] J. M. Wessels , A. M. Felker , H. A. Dupont , and C. Kaushic , “The Relationship Between Sex Hormones, the Vaginal Microbiome and Immunity in HIV‐1 Susceptibility in Women,” Disease Models & Mechanisms 11 (2018): dmm035147. 30154116 10.1242/dmm.035147 PMC6177003

[89] K. Peebles , F. M. Kiweewa , T. Palanee‐Phillips , et al., “Elevated Risk of Bacterial Vaginosis Among Users of the Copper Intrauterine Device: A Prospective Longitudinal Cohort Study,” Clinical Infectious Diseases 73 (2020): 513–520. 10.1093/cid/ciaa703 PMC8326546 32505132

[90] N. Rahman , M. F. Mian , A. Nazli , and C. Kaushic , “Human Vaginal Microbiota Colonization Is Regulated by Female Sex Hormones in a Mouse Model,” Frontiers in Cellular and Infection Microbiology 13 (2023): 1307451. 38156321 10.3389/fcimb.2023.1307451 PMC10753781

[91] C. Kaushic , K. L. Roth , V. Anipindi , and F. Xiu , “Increased Prevalence of Sexually Transmitted Viral Infections in Women: The Role of Female Sex Hormones in Regulating Susceptibility and Immune Responses,” Journal of Reproductive Immunology 88 (2011): 204–209. 21296427 10.1016/j.jri.2010.12.004

[92] R. Stricker , R. Eberhart , M. C. Chevailler , F. A. Quinn , P. Bischof , and R. Stricker , “Establishment of Detailed Reference Values for Luteinizing Hormone, Follicle Stimulating Hormone, Estradiol, and Progesterone During Different Phases of the Menstrual Cycle on the Abbott ARCHITECT Analyzer,” Clinical Chemistry and Laboratory Medicine 44 (2006): 883–887. 16776638 10.1515/CCLM.2006.160

[93] H. Kaur , M. Merchant , M. M. Haque , and S. S. Mande , “Crosstalk Between Female Gonadal Hormones and Vaginal Microbiota Across Various Phases of Women's Gynecological Lifecycle,” Frontiers in Microbiology 11 (2020): 551. 32296412 10.3389/fmicb.2020.00551 PMC7136476

[94] H. Zhu and X. Zhang , “Correlation Analysis Between Vaginal Microbiota and Quality of Life Across Different Female Life Stages,” BMC Microbiology 26 (2026): 360. 41803680 10.1186/s12866-026-04908-w PMC13085509

[95] R. C. Wira and V. J. Fahey , “A New Strategy to Understand How HIV Infects Women: Identification of a Window of Vulnerability During the Menstrual Cycle,” AIDS 22 (2008): 1909–1917. 18784454 10.1097/QAD.0b013e3283060ea4 PMC2647143

[96] C. R. Wira , M. Rodriguez‐Garcia , and M. V. Patel , “The Role of Sex Hormones in Immune Protection of the Female Reproductive Tract,” Nature Reviews Immunology 15 (2015): 217–230. 10.1038/nri3819 PMC4716657 25743222

[97] R. Amsel , P. A. Totten , C. A. Spiegel , K. C. Chen , D. Eschenbach , and K. K. Holmes , “Nonspecific Vaginitis. Diagnostic Criteria and Microbial and Epidemiologic Associations,” American Journal of Medicine 74 (1983): 14–22. 6600371 10.1016/0002-9343(83)91112-9

[98] S. Hillier , R. Nugent , D. Eschenbach , et al., “Association Between Bacterial Vaginosis and Preterm Delivery of a Low‐Birth‐Weight Infant,” NEJM 333 (1995): 1737–1742. 7491137 10.1056/NEJM199512283332604

[99] D. H. Watts , M. A. Krohn , S. L. Hillier , and D. A. Eschenbach , “Bacterial Vaginosis as a Risk Factor for Post‐Cesarean Endometritis,” Obstetrics and Gynecology 75 (1990): 52–58. 2296423

[100] H. C. Wiesenfeld , S. L. Hillier , M. A. Krohn , et al., “Lower Genital Tract Infection and Endometritis: Insight Into Subclinical Pelvic Inflammatory Disease,” Obstetrics and Gynecology 100 (2002): 456–463. 12220764 10.1016/s0029-7844(02)02118-x

[101] R. H. Beigi , M. H. Yudin , L. Cosentino , L. A. Meyn , and S. L. Hillier , “Cytokines, Pregnancy, and Bacterial Vaginosis: Comparison of Levels of Cervical Cytokines in Pregnant and Nonpregnant Women With Bacterial Vaginosis,” Journal of Infectious Diseases 196 (2007): 1355–1360. 17922400 10.1086/521628

[102] M. A. Elovitz , P. Gajer , V. Riis , et al., “Cervicovaginal Microbiota and Local Immune Response Modulate the Risk of Spontaneous Preterm Delivery,” Nature Communications 10 (2019): 1305. 10.1038/s41467-019-09285-9 PMC6428888 30899005

[103] S. Tuddenham , J. Ravel , and J. M. Marrazzo , “Protection and Risk: Male and Female Genital Microbiota and Sexually Transmitted Infections,” Journal of Infectious Diseases 223 (2021): S222–S235. 33576776 10.1093/infdis/jiaa762 PMC8206802

[104] D. E. O'Hanlon , P. Gajer , R. M. Brotman , and J. Ravel , “Asymptomatic Bacterial Vaginosis Is Associated With Depletion of Mature Superficial Cells Shed From the Vaginal Epithelium,” Frontiers in Cellular And Infection Microbiology 10 (2020): 106. 32211347 10.3389/fcimb.2020.00106 PMC7076050

[105] C. S. Bradshaw , E. L. Plummer , C. A. Muzny , et al., “Bacterial Vaginosis,” Nature Reviews Disease Primers 11 (2025): 43. 10.1038/s41572-025-00626-1 40537474

[106] A. C. Roxby , N. R. Mugo , L. M. Oluoch , et al., “Low Prevalence of Bacterial Vaginosis in Kenyan Adolescent Girls and Rapid Incidence After First Sex,” American Journal of Obstetrics and Gynecology 229 (2023): 282.e1–282.e11. 10.1016/j.ajog.2023.06.044 PMC10530291 37391005

[107] L. Ratten , E. Plummer , G. Murray , et al., “Sex Is Associated With the Persistence of Non‐Optimal Vaginal Microbiota Following Treatment for Bacterial Vaginosis: A Prospective Cohort Study,” BJOG 128 (2021): 756–767. 33480468 10.1111/1471-0528.16430

[108] E. L. Plummer , L. A. Vodstrcil , J. A. Danielewski , et al., “Combined Oral and Topical Antimicrobial Therapy for Male Partners of Women With Bacterial Vaginosis: Acceptability, Tolerability and Impact on the Genital Microbiota of Couples—A Pilot Study,” PLoS ONE 13 (2018): e0190199. 29293559 10.1371/journal.pone.0190199 PMC5749747

[109] E. L. Plummer , L. A. Vodstrcil , M. Doyle , et al., “A Prospective, Open‐Label Pilot Study of Concurrent Male Partner Treatment for Bacterial Vaginosis,” MBio 12 (2021): e0232321. 34663095 10.1128/mBio.02323-21 PMC8524345

[110] L. A. Vodstrcil , E. L. Plummer , C. K. Fairley , et al., “Male‐Partner Treatment to Prevent Recurrence of Bacterial Vaginosis,” New England Journal of Medicine 392 (2025): 947–957. 40043236 10.1056/NEJMoa2405404

[111] V. Joag , O. Obila , P. Gajer , et al., “Impact of Standard Bacterial Vaginosis Treatment on the Genital Microbiota, Immune Milieu, and Ex Vivo Human Immunodeficiency Virus Susceptibility,” Clinical Infectious Diseases 68 (2018): 1675–1683. 10.1093/cid/ciy762 PMC6495022 30407498

[112] S. Y. Doerflinger , A. L. Throop , and H. K. MM , “Bacteria in the Vaginal Microbiome Alter the Innate Immune Response and Barrier Properties of the Human Vaginal Epithelia in a Species‐Specific Manner,” Journal of Infectious Diseases 209 (2014): 1989–1999. 24403560 10.1093/infdis/jiu004

[113] A. Alisoltani , M. T. Manhanzva , M. Potgieter , et al., “Microbial Function and Genital Inflammation in Young South African Women at High Risk of HIV Infection,” Microbiome 8 (2020): 165. 33220709 10.1186/s40168-020-00932-8 PMC7679981

[114] A. Decout , I. Krasias , L. Roberts , et al., “Lactobacillus Crispatus S‐Layer Proteins Modulate Innate Immune Response and Inflammation in the Lower Female Reproductive Tract,” Nature Communications 15 (2024): 10879. 10.1038/s41467-024-55233-7 PMC11685708 39737998

[115] A. C. Hearps , D. Tyssen , D. Srbinovski , et al., “Vaginal Lactic Acid Elicits an Anti‐Inflammatory Response From Human Cervicovaginal Epithelial Cells and Inhibits Production of Pro‐Inflammatory Mediators Associated With HIV Acquisition,” Mucosal Immunology 10 (2017): 1480–1490. 28401934 10.1038/mi.2017.27

[116] E. K. Libby , K. E. Pascal , E. Mordechai , M. E. Adelson , and J. P. Trama , “Atopobium Vaginae Triggers an Innate Immune Response in an in Vitro Model of Bacterial Vaginosis,” Microbes and Infection 10 (2008): 439–446. 18403235 10.1016/j.micinf.2008.01.004

[117] R. N. Fichorova , A. B. Onderdonk , H. Yamamoto , et al., “Maternal Microbe‐Specific Modulation of Inflammatory Response in Extremely Low‐Gestational‐age Newborns,” MBio 2 (2011): e00280–10. 10.1128/mBio.00280-10 PMC3025357 21264056

[118] D. J. Delgado‐Diaz , D. Tyssen , J. A. Hayward , R. Gugasyan , A. C. Hearps , and G. Tachedjian , “Distinct Immune Responses Elicited From Cervicovaginal Epithelial Cells by Lactic Acid and Short Chain Fatty Acids Associated With Optimal and Non‐Optimal Vaginal Microbiota,” Frontiers in Cellular and Infection Microbiology 9 (2020): 446. 31998660 10.3389/fcimb.2019.00446 PMC6965070

[119] T. M. Nelson , J.‐L. C. Borgogna , R. M. Brotman , J. Ravel , S. T. Walk , and C. J. Yeoman , “Vaginal Biogenic Amines: Biomarkers of Bacterial Vaginosis or Precursors to Vaginal Dysbiosis?,” Frontiers in Physiology 6 (2015): 253–253. 26483694 10.3389/fphys.2015.00253 PMC4586437

[120] J. C. Borgogna , M. D. Shardell , S. G. Grace , et al., “Biogenic Amines Increase the Odds of Bacterial Vaginosis and Affect the Growth of and Lactic Acid Production by Vaginal Lactobacillus spp,” Applied and Environmental Microbiology 87 (2021): e03068–20. 10.1128/AEM.03068-20 PMC8117770 33674429

[121] A. Joseph , L. Anton , Y. Guan , et al., “Extracellular Vesicles From Vaginal Gardnerella Vaginalis and Mobiluncus Mulieris Contain Distinct Proteomic Cargo and Induce Inflammatory Pathways,” NPJ Biofilms and Microbiomes 10 (2024): 28. 38514622 10.1038/s41522-024-00502-y PMC10957959

[122] L. Chen , J. Li , and B. Xiao , “The Role of Sialidases in the Pathogenesis of Bacterial Vaginosis and Their Use as a Promising Pharmacological Target in Bacterial Vaginosis,” Frontiers in Cellular and Infection Microbiology 14 (2024): 1367233. 38495652 10.3389/fcimb.2024.1367233 PMC10940449

[123] J. H. Elnaggar , J. W. Lammons , C. M. Taylor , et al., “Characterization of Vaginal Microbial Community Dynamics in the Pathogenesis of Incident Bacterial Baginosis,” Sexually Transmitted Diseases 50 (2023): 523–530. 37074327 10.1097/OLQ.0000000000001821 PMC10512881

[124] Y. Wang , L. Noël‐Romas , M. Perner , et al., “Non‐Lactobacillus‐Dominant and Polymicrobial Vaginal Microbiomes Are More Common in Younger South African Women and Predictive of Increased Risk of Human Immunodeficiency Virus Acquisition,” Clinical Infectious Diseases 76 (2022): 1372–1381. 10.1093/cid/ciac938 PMC10110272 36504254

[125] C. Kenyon , R. Colebunders , and T. Crucitti , “The Global Epidemiology of Bacterial Vaginosis: A Systematic Review,” American Journal of Obstetrics and Gynecology 209 (2013): 505–523. 23659989 10.1016/j.ajog.2013.05.006

[126] C. Balle , K. Lennard , S. Dabee , et al., “Endocervical and Vaginal Microbiota in South African Adolescents With Asymptomatic Chlamydia trachomatis infection,” Scientific Reports 8 (2018): 11109. 30038262 10.1038/s41598-018-29320-x PMC6056523

[127] S. Srinivasan , B. A. Richardson , J. M. Wallis , et al., “Vaginal Bacteria and Proinflammatory Host Immune Mediators as Biomarkers of Human Immunodeficiency Virus Acquisition Risk Among African Women,” Journal of Infectious Diseases 230 (2024): 1444–1455. 39248500 10.1093/infdis/jiae406 PMC11646615

[128] R. S. McClelland , J. R. Lingappa , S. Srinivasan , et al., “Evaluation of the Association Between the Concentrations of Key Vaginal Bacteria and the Increased Risk of HIV Acquisition in African Women From Five Cohorts: A Nested Case‐Control Study,” Lancet Infectious Diseases 18 (2018): 554–564. 29396006 10.1016/S1473-3099(18)30058-6 PMC6445552

[129] H. Borgdorff , E. Tsivtsivadze , R. Verhelst , et al., “Lactobacillus‐Dominated Cervicovaginal Microbiota Associated With Reduced HIV/STI Prevalence and Genital HIV Viral Load in African Women,” ISME journal 8 (2014): 1781–1793. 24599071 10.1038/ismej.2014.26 PMC4139719

[130] D. Vitali , J. M. Wessels , and C. Kaushic , “Role of Sex Hormones and the Vaginal Microbiome in Susceptibility and Mucosal Immunity to HIV‐1 in the Female Genital Tract,” AIDS Research Therapy 14 (2017): 39–39. 28893284 10.1186/s12981-017-0169-4 PMC5594427

[131] H. Borgdorff , R. Gautam , S. D. Armstrong , et al., “Cervicovaginal Microbiome Dysbiosis Is Associated With Proteome Changes Related to Alterations of the Cervicovaginal Mucosal Barrier,” Mucosal Immunology 9 (2016): 621–633. 26349657 10.1038/mi.2015.86

[132] A. S. Zevin , I. Y. Xie , K. Birse , et al., “Microbiome Composition and Function Drives Wound‐Healing Impairment in the Female Genital Tract,” Plos Pathogens 12 (2016): e1005889. 27656899 10.1371/journal.ppat.1005889 PMC5033340

[133] L. Masson , J. Wilson , A. S. Amir Hamzah , G. Tachedjian , and M. Payne , “Advances in Mass Spectrometry Technologies to Characterize Cervicovaginal Microbiome Functions That Impact Spontaneous Preterm Birth,” American Journal of Reproductive Immunology 90 (2023): e13750. 37491925 10.1111/aji.13750

[134] J. B. Holm , M. T. France , P. Gajer , et al., “Integrating Compositional and Functional Content to Describe Vaginal Microbiomes in Health and Disease,” Microbiome 11 (2023): 259. 38031142 10.1186/s40168-023-01692-x PMC10688475

[135] M. T. Manhanzva , A. G. Abrahams , H. Gamieldien , et al., “Inflammatory and Antimicrobial Properties Differ Between Vaginal Lactobacillus Isolates From South African Women With Non‐Optimal Versus Optimal Microbiota,” Scientific Reports 10 (2020): 6196. 32277092 10.1038/s41598-020-62184-8 PMC7148372

[136] A. R. Berard , D. K. Brubaker , K. Birse , et al., “Vaginal Epithelial Dysfunction Is Mediated by the Microbiome, Metabolome, and mTOR Signaling,” Cell reports 42 (2023): 112474. 37149863 10.1016/j.celrep.2023.112474 PMC10242450

[137] S. Srinivasan , M. T. Morgan , T. L. Fiedler , et al., “Metabolic Signatures of Bacterial Vaginosis,” MBio 6 (2015): e00204–15. 10.1128/mBio.00204-15 PMC4453549 25873373

[138] J. L. McCarville , G. Y. Chen , V. D. Cuevas , K. Troha , and J. S. Ayres , “Microbiota Metabolites in Health and Disease,” Annual Review of Immunology 38 (2020): 147–170. 10.1146/annurev-immunol-071219-125715 32340573

[139] E. Amabebe and D. O. C. Anumba , “Female Gut and Genital Tract Microbiota‐Induced Crosstalk and Differential Effects of Short‐Chain Fatty Acids on Immune Sequelae,” Frontiers in Immunology 11 (2020): 2184. 33013918 10.3389/fimmu.2020.02184 PMC7511578

[140] T. Suzuki , S. Yoshida , and H. Hara , “Physiological Concentrations of Short‐Chain Fatty Acids Immediately Suppress Colonic Epithelial Permeability,” British Journal of Nutrition 100 (2008): 297–305. 18346306 10.1017/S0007114508888733

[141] I. Schwecht , A. Nazli , B. Gill , and C. Kaushic , “Lactic Acid Enhances Vaginal Epithelial Barrier Integrity and Ameliorates Inflammatory Effects of Dysbiotic Short Chain Fatty Acids and HIV‐1,” Scientific Reports 13 (2023): 20065. 37973920 10.1038/s41598-023-47172-y PMC10654711

[142] G. Tachedjian , D. E. O'Hanlon , and J. Ravel , “The Implausible “in Vivo” Role of Hydrogen Peroxide as an Antimicrobial Factor Produced by Vaginal Microbiota,” Microbiome 6 (2018): 29. 29409534 10.1186/s40168-018-0418-3 PMC5801833

[143] Z. Gong , Y. Luna , P. Yu , and H. Fan , “Lactobacilli Inactivate Chlamydia Trachomatis Through Lactic Acid but Not H2O2,” PLoS ONE 9 (2014): e107758. 25215504 10.1371/journal.pone.0107758 PMC4162611

[144] A. Aroutcheva , D. Gariti , M. Simon , et al., “Defense Factors of Vaginal Lactobacilli,” American Journal of Obstetrics and Gynecology 185 (2001): 375–379. 11518895 10.1067/mob.2001.115867

[145] D. E. O'Hanlon , T. R. Moench , and R. A. Cone , “Vaginal pH and Microbicidal Lactic Acid When Lactobacilli Dominate the Microbiota,” PLoS ONE 8 (2013): e80074–e80074. 24223212 10.1371/journal.pone.0080074 PMC3819307

[146] E. R. Boskey , R. A. Cone , K. J. Whaley , and T. R. Moench , “Origins of Vaginal Acidity: High D/L Lactate Ratio Is Consistent With Bacteria Being the Primary Source,” Human Reproduction 16 (2001): 1809–1813. 11527880 10.1093/humrep/16.9.1809

[147] J. Wertz , N. Isaacs‐Cosgrove , C. Holzman , and T. L. Marsh , “Temporal Shifts in Microbial Communities in Nonpregnant African‐American Women With and Without Bacterial Vaginosis,” Interdisciplinary Perspectives on Infectious Diseases 2008 (2008): 181253. 19277101 10.1155/2008/181253 PMC2648625

[148] M. Aldunate , D. Tyssen , A. Johnson , et al., “Vaginal Concentrations of Lactic Acid Potently Inactivate HIV,” Journal of Antimicrobial Chemotherapy 68 (2013): 2015–2025. 23657804 10.1093/jac/dkt156 PMC3743514

[149] D. E. O'Hanlon , R. A. Cone , and T. R. Moench , “Vaginal pH Measured in Vivo: Lactobacilli Determine pH and Lactic Acid Concentration,” BMC Microbiology 19 (2019): 13. 30642259 10.1186/s12866-019-1388-8 PMC6332693

[150] S. S. Olmsted , K. V. Khanna , E. M. Ng , et al., “Low pH Immobilizes and Kills Human Leukocytes and Prevents Transmission of Cell‐Associated HIV in a Mouse Model,” BMC Infectious Diseases [Electronic Resource] 5 (2005): 79. 16194280 10.1186/1471-2334-5-79 PMC1262719

[151] R. A. Cone , “Vaginal Microbiota and Sexually Transmitted Infections That May Influence Transmission of Cell‐Associated HIV,” Journal of Infectious Diseases 210, no. 3 (2014): S616–21. 10.1093/infdis/jiu459 PMC4303075 25414415

[152] M. A. Graver and J. J. Wade , “The Role of Acidification in the Inhibition of Neisseria Gonorrhoeae by Vaginal Lactobacilli During anaerobic Growth,” Annals of Clinical Microbiology and Antimicrobials 10 (2011): 8–8. 21329492 10.1186/1476-0711-10-8 PMC3045876

[153] E. V. Valore , C. H. Park , S. L. Igreti , and T. Ganz , “Antimicrobial Components of Vaginal Fluid,” American Journal of Obstetrics and Gynecology 187 (2002): 561–568. 12237628 10.1067/mob.2002.125280

[154] P. Ellenberg , D. Tyssen , and J. Wilson , “Inhibitory Activity of Lactic Acid Isomers Against L. iners and BV‐associated Vaginal Bacteria to Prevent HIV Transmission,” in Abstracts from HIVR4P 2024, the 5th HIV Research for Prevention Conference, 6 – 10 October, (John Wiley & Sons LTD), e26351.

[155] D. J. Delgado‐Diaz , B. Jesaveluk , J. A. Hayward , et al., “Lactic Acid From Vaginal Microbiota Enhances Cervicovaginal Epithelial Barrier Integrity by Promoting Tight Junction Protein Expression,” Microbiome 10 (2022): 141. 36045402 10.1186/s40168-022-01337-5 PMC9429363

[156] B. Jesaveluk , “The impact of the vaginal environment on epithelial barrier function and HIV translocation,” (Monash University, 2024), 10.26180/26888893.

[157] C. S. Bradshaw and R. M. Brotman , “Making Inroads Into Improving Treatment of Bacterial Vaginosis–Striving for Long‐Term Cure,” BMC Infectious Diseases [Electronic Resource] 15 (2015): 292. 26219949 10.1186/s12879-015-1027-4 PMC4518586

[158] J. D. Sobel , “Recurrent Bacterial Vaginosis, Relapse or Reinfection: The Role of Sexual Transmission,” BJOG 128 (2021): 768–768. 33241645 10.1111/1471-0528.16471

[159] E. L. Plummer , A. M. Sfameni , L. A. Vodstrcil , et al., “Prevotella and Gardnerella Are Associated With Treatment Failure Following First‐Line Antibiotics for Bacterial Vaginosis,” Journal of Infectious Diseases 228 (2023): 646–656. 37427495 10.1093/infdis/jiad261 PMC10469350

[160] J. D. C. Ross , S. Thandi , C. Brittain , J. Kai , and F. Griffiths , “Acceptability of and Treatment Preferences for Recurrent Bacterial Vaginosis—Topical Lactic Acid Gel or Oral Metronidazole Antibiotic: Qualitative Findings From the VITA Trial,” PLoS ONE 14 (2019): e0224964. 31730666 10.1371/journal.pone.0224964 PMC6857901

[161] L. Armstrong‐Buisseret , C. Brittain , J. Kai , et al., “Lactic Acid Gel Versus Metronidazole for Recurrent Bacterial Vaginosis in Women Aged 16 Years and Over: The VITA RCT,” Health Technology Assessment 26 (2022): 1–170. 10.3310/ZZKH4176 35057905

[162] E. Armstrong , A. Hemmerling , S. Miller , et al., “Sustained Effect of LACTIN‐V (Lactobacillus Crispatus CTV‐05) on Genital Immunology Following Standard Bacterial Vaginosis Treatment: Results From a Randomised, Placebo‐Controlled Trial,” Lancet Microbe 3 (2022): e435–e442. 35659905 10.1016/S2666-5247(22)00043-X PMC9188188

[163] J. Ravel , S. Simmons , E. G. Jaswa , et al., “Impact of a Multi‐Strain L. Crispatus‐Based Vaginal Synbiotic on the Vaginal Microbiome: A Randomized Placebo‐Controlled Trial,” NPJ Biofilms and Microbiomes 11 (2025): 158. 40783570 10.1038/s41522-025-00788-6 PMC12335476

[164] C. R. Cohen , M. R. Wierzbicki , A. L. French , et al., “Randomized Trial of LACTIN‐V to Prevent Recurrence of Bacterial Vaginosis,” New England Journal of Medicine 382 (2020): 1906–1915. 32402161 10.1056/NEJMoa1915254 PMC7362958

[165] D. Potloane , L. Symul , S. Ngcapu , et al., “VIBRANT: A Phase 1 Randomized Trial of Multi‐Strain Vaginal L. Crispatus Live Biotherapeutic Products in People With Bacterial Vaginosis,” Cell Host & Microbe 34 (2026): 751–760.e5. 41856107 10.1016/j.chom.2026.02.016

[166] E. L. Plummer , C. S. Bradshaw , M. Doyle , et al., “Lactic Acid‐Containing Products for Bacterial Vaginosis and Their Impact on the Vaginal Microbiota: A Systematic Review,” PLoS ONE 16 (2021): e0246953. 33571286 10.1371/journal.pone.0246953 PMC7877752

[167] S. M. Bloom , N. A. Mafunda , B. M. Woolston , et al., “Cysteine Dependence of Lactobacillus Iners Is a Potential Therapeutic Target for Vaginal Microbiota Modulation,” Nature Microbiology 7 (2022): 434–450. 10.1038/s41564-022-01070-7 PMC10473153 35241796

[168] M. Zhu , M. W. Frank , C. D. Radka , et al., “Vaginal Lactobacillus Fatty Acid Response Mechanisms Reveal a Metabolite‐Targeted Strategy for Bacterial Vaginosis Treatment,” Cell 187 (2024): 5413–5430.e29. 39163861 10.1016/j.cell.2024.07.029 PMC11429459

[169] A. Abbasi , L. Aghebati‐Maleki , and A. Homayouni‐Rad , “The Promising Biological Role of Postbiotics Derived From Probiotic Lactobacillus Species in Reproductive Health,” Critical Reviews in Food Science and Nutrition 62 (2021): 8829–8841. 34152234 10.1080/10408398.2021.1935701

[170] M. S. Kwon and H. K. Lee , “Host and Microbiome Interplay Shapes the Vaginal Microenvironment,” Frontiers in Immunology 13 (2022): 919728. 35837395 10.3389/fimmu.2022.919728 PMC9273862

[171] S. Wu , L. W. Hugerth , I. Schuppe‐Koistinen , and J. Du , “The Right Bug in the Right Place: Opportunities for Bacterial Vaginosis Treatment,” NPJ Biofilms and Microbiomes 8 (2022): 34. 35501321 10.1038/s41522-022-00295-y PMC9061781

[172] A. Hemmerling , C. M. Mitchell , S. Demby , et al., “Effect of the Vaginal Live Biotherapeutic LACTIN‐V (Lactobacillus Crispatus CTV‐05) on Vaginal Microbiota and Genital Tract Inflammation Among Women at High Risk of HIV Acquisition in South Africa: A Phase 2, Randomised, Placebo‐Controlled Trial,” Lancet Microbe 6 (2025): 101037. 40194532 10.1016/j.lanmic.2024.101037 PMC12173475

[173] E. Bayar , D. A. MacIntyre , L. Sykes , et al., “Safety, Tolerability, and Acceptability of Lactobacillus Crispatus CTV‐05 (LACTIN‐V) in Pregnant Women at High‐Risk of Preterm Birth,” Beneficial Microbes 14 (2023): 45–56. 36815494 10.3920/BM2022.0084

[174] J. A. Fernández‐Romero , C. Deal , B. C. Herold , et al., “Multipurpose Prevention Technologies: The Future of HIV and STI Protection,” Trends in Microbiology 23 (2015): 429–436. 25759332 10.1016/j.tim.2015.02.006 PMC4490993

[175] B. Andersch , L. Forssman , K. Lincoln , and P. Torstensson , “Treatment of Bacterial Vaginosis With an Acid Cream: A Comparison Between the Effect of Lactate‐gel and Metronidazole,” Gynecologic and Obstetric Investigation 21 (1986): 19–25. 3485071 10.1159/000298923

[176] A. J. Boeke , J. H. Dekker , J. T. van Eijk , P. J. Kostense , and P. D. Bezemer , “Effect of Lactic Acid Suppositories Compared With Oral Metronidazole and Placebo in Bacterial Vaginosis: A Randomised Clinical Trial,” Genitourinary Medicine 69 (1993): 388. 8244360 10.1136/sti.69.5.388 PMC1195125

[177] J. A. Simoes , L. G. Bahamondes , R. P. S. Camargo , et al., “A Pilot Clinical Trial Comparing an Acid‐Buffering Formulation (ACIDFORM gel) With Metronidazole Gel for the Treatment of Symptomatic Bacterial Vaginosis,” British Journal of Clinical Pharmacology 61 (2006): 211–217. 16433875 10.1111/j.1365-2125.2005.02550.x PMC1884999

[178] J. Robertsson , “A New Effective, User‐Friendly Bacterial Vaginosis Treatment: A Randomized Multicenter Open‐Label Parallel‐Group Two‐Part Study With a Novel Sustained‐Release Pessary Containing Oligomeric Lactic Acid,” Journal of Infectious & Non Infectious Diseases 1 (2015): 1–12.

[179] C. van der Veer , S. M. Bruisten , R. van Houdt , et al., “Effects of an Over‐the‐Counter Lactic‐Acid Containing Intra‐Vaginal Douching Product on the Vaginal Microbiota,” BMC Microbiology 19 (2019): 168. 31345159 10.1186/s12866-019-1545-0 PMC6659218

[180] C. Gottschick , Z.‐L. Deng , M. Vital , et al., “Treatment of Biofilms in Bacterial Vaginosis by an Amphoteric Tenside Pessary‐Clinical Study and Microbiota Analysis,” Microbiome 5 (2017): 119. 28903767 10.1186/s40168-017-0326-y PMC5598074

[181] L. Armstrong‐Buisseret , C. Brittain , M. David , et al., “Metronidazole Versus Lactic Acid for Treating Bacterial Vaginosis (VITA): Protocol for a Randomised Controlled Trial to Assess the Clinical and Cost Effectiveness of Topical Lactic Acid Gel for Treating Second and Subsequent Episodes of Bacterial Vaginosis,” Trials 20 (2019): 648. 31775859 10.1186/s13063-019-3731-7 PMC6880606

[182] J. D. C. Ross , C. Brittain , J. Anstey Watkins , et al., “Intravaginal Lactic Acid Gel Versus Oral Metronidazole for Treating Women With Recurrent Bacterial Vaginosis: The VITA Randomised Controlled Trial,” BMC Womens Health 23 (2023): 241. 37161454 10.1186/s12905-023-02303-5 PMC10169495

[183] A. B. Campaner , A. C. A. Rosário Sica , F. S. d' Avila Curi , G. Marchetti , G. S. Dias , and B. L. Teixeira , “In Vivo Assessment of the Effect of Gel Containing Lactic Acid and Glycogen on Vaginal Microbiota and pH of Asymptomatic Women of Reproductive Age,” PLoS ONE 20 (2025): e0321737. 40273081 10.1371/journal.pone.0321737 PMC12021183

[184] D. Tyssen , A. C. Hearps , K. Guntur , et al., “The Impact of Over‐the‐Counter Lactic Acid Containing Vaginal Gels on the Integrity an Inflammatory State of the Vaginal Epithelium in Vitro,” Frontiers in Reproductive Health 4 (2022): 915948. 36303665 10.3389/frph.2022.915948 PMC9580709

[185] J. Perrin , G. E. Costin , S. Ayehunie , et al., “Using a Reconstructed Human Vaginal Epithelium Model to Assess Irritation: A Proof‐of‐Concept Study Supporting Regulatory Qualification of the Method for Use With Personal Lubricants,” Toxicology in Vitro 112 (2026): 106198. 41548851 10.1016/j.tiv.2026.106198

[186] M. Hu , T. Zhou , C. S. Dezzutti , and L. C. Rohan , “The Effect of Commonly Used Excipients on the Epithelial Integrity of Human Cervicovaginal Tissue,” Aids Research and Human Retroviruses 32 (2016): 992–1004. 27611224 10.1089/aid.2016.0014 PMC5067878

